# The Role of AI in Cardiovascular Event Monitoring and Early Detection: Scoping Literature Review

**DOI:** 10.2196/64349

**Published:** 2025-03-06

**Authors:** Luis B Elvas, Ana Almeida, Joao C Ferreira

**Affiliations:** 1 Department of Logistics, Molde University College, Molde, Norway; 2 INESC INOV Rua Alves Redol, Lisbon, Portugal; 3 Instituto Universitário de Lisboa (ISCTE-IUL), ISTAR, Lisboa, Portugal

**Keywords:** artificial intelligence, machine learning, cardiovascular diseases, cardiovascular events, health care, monitoring, early detection, AI, cardiovascular, literature review, medical data, detect, patient outcomes, neural network, ML model, mobile phone

## Abstract

**Background:**

Artificial intelligence (AI) has shown exponential growth and advancements, revolutionizing various fields, including health care. However, domain adaptation remains a significant challenge, as machine learning (ML) models often need to be applied across different health care settings with varying patient demographics and practices. This issue is critical for ensuring effective and equitable AI deployment. Cardiovascular diseases (CVDs), the leading cause of global mortality with 17.9 million annual deaths, encompass conditions like coronary heart disease and hypertension. The increasing availability of medical data, coupled with AI advancements, offers new opportunities for early detection and intervention in cardiovascular events, leveraging AI’s capacity to analyze complex datasets and uncover critical patterns.

**Objective:**

This review aims to examine AI methodologies combined with medical data to advance the intelligent monitoring and detection of CVDs, identifying areas for further research to enhance patient outcomes and support early interventions.

**Methods:**

This review follows the PRISMA (Preferred Reporting Items for Systematic Reviews and Meta-Analyses) methodology to ensure a rigorous and transparent literature review process. This structured approach facilitated a comprehensive overview of the current state of research in this field.

**Results:**

Through the methodology used, 64 documents were retrieved, of which 40 documents met the inclusion criteria. The reviewed papers demonstrate advancements in AI and ML for CVD detection, classification, prediction, diagnosis, and patient monitoring. Techniques such as ensemble learning, deep neural networks, and feature selection improve prediction accuracy over traditional methods. ML models predict cardiovascular events and risks, with applications in monitoring via wearable technology. The integration of AI in health care supports early detection, personalized treatment, and risk assessment, possibly improving the management of CVDs.

**Conclusions:**

The study concludes that AI and ML techniques can improve the accuracy of CVD classification, prediction, diagnosis, and monitoring. The integration of multiple data sources and noninvasive methods supports continuous monitoring and early detection. These advancements help enhance CVD management and patient outcomes, indicating the potential for AI to offer more precise and cost-effective solutions in health care.

## Introduction

Artificial intelligence (AI) has revolutionized various aspects of our lives, showing exponential growth and advancements. The ubiquity of AI and its influence on nearly every facet of human life, from business and entertainment to technology, has not yet been fully realized in the field of medicine [1]. Despite these strides, machine learning (ML) in health care faces significant challenges, particularly in the realm of domain adaptation, where the goal is to apply ML models developed in one context to different but related health care settings. This is particularly crucial in health care, where the variability in patient demographics, disease prevalence, and health care practices across different regions and populations can affect the performance and reliability of ML models. Addressing these challenges is essential for ensuring that AI-driven technologies can be effectively and equitably applied across diverse health care environments [2].

The increasing availability of medical data, combined with advancements in AI techniques, creates new opportunities for developing intelligent systems that can assist health professionals in the early detection and rapid intervention of cardiovascular events [3]. For instance, studies like Elvas et al [4] have demonstrated the capability of predictive models to anticipate myocardial ischemia incidents, enabling timely interventions and improving patient outcomes. One key advantage of AI in this context is its ability to analyze large volumes of complex data, uncovering patterns and correlations that might be missed by analysts [5,6]. This capability could be especially significant in the context of cardiology, the specific subject of our study, helping to provide a more comprehensive understanding of a patient’s cardiovascular health.

Cardiovascular diseases (CVDs) remain the leading cause of mortality worldwide [7]. Globally, CVDs account for approximately 17.9 million deaths annually, representing 32% of all global deaths [8]. These conditions encompass a range of disorders affecting the heart and blood vessels, including coronary heart disease, rheumatic heart disease, congenital heart defects, and hypertension. The total number of CVD deaths and years of life lost are likely to increase as a result of population growth and aging [9], underscoring the critical need for effective methods to monitor and diagnose these conditions early.

The primary objective of this review is to address these gaps by conducting a comprehensive review of studies focusing on AI methodologies, in conjunction with medical data, that contributed to knowledge and progress in the intelligent monitoring, detection, prediction, and diagnosis of these kinds of diseases. Specially, this review aims to examine and synthesize current research on the application of AI and ML methodologies in CVD management. The study seeks to identify and evaluate various AI-driven approaches, including deep learning (DL) and ensemble methods, that have shown promise in improving the accuracy of CVD classification, risk assessment, and patient monitoring. Additionally, the review aims to highlight the potential of these technologies in enabling diverse treatment strategies, supporting health care professionals’ decision-making processes, and ultimately enhancing patient outcomes. By assessing the current state of research, the review also intends to identify challenges, limitations, and areas for further investigation to guide future advancements in AI-driven cardiovascular care.

Despite the comprehensive nature of this review, it is important to acknowledge certain limitations and challenges that affected the search process. The review is confined to papers published between 2020 and 2024, which is a narrow year range, however, no papers were found outside of that range using that selected search query. Additionally, the search was limited to English-language publications, which may have resulted in the omission of valuable research published in other languages. One significant challenge is the variability in study quality across the selected literature. The rapidly evolving nature of AI and ML techniques in health care means that methodologies and reporting standards may vary considerably between studies, potentially affecting the comparability and generalizability of findings. Additionally, the integration of diverse AI methodologies presents a challenge in synthesizing results, as different approaches may have varying levels of interpretability and clinical applicability. The heterogeneity in data sources, sample sizes, and outcome measures across studies may also complicate the process of drawing cohesive conclusions. Furthermore, the field’s fast-paced development means that some cutting-edge methodologies might be underrepresented in peer-reviewed literature at the time of this review. These limitations should be considered when interpreting the findings and their implications for clinical practice and future research directions in AI-driven cardiovascular care.

In summary, this literature review highlights the transformative potential of AI in health care, particularly in the domain of CVD. It underscored the significant global impact of CVD and the urgent need for improved methods of monitoring, detection, and early intervention. The review aims to explore how AI and ML techniques, when combined with extensive medical data, can advance our approach to managing cardiovascular health. By examining current methodologies and their practical implications, there were identified areas for further research and development. As health care evolves, incorporating intelligent systems may improve patient outcomes and support early interventions [10]. The potential for AI to transform cardiovascular health care is immense, offering the promise of more accurate diagnoses, personalized treatment plans, and improved patient outcomes.

## Methods

The PRISMA (Preferred Reporting Items for Systematic Reviews and Meta-Analyses) methodology assisted the search by providing an organized structure and clear steps. Originally developed for medical research, PRISMA provides evidence-based guidelines to help authors transparently report their review process, methods, and findings [11].

This methodology ensures a rigorous literature review, providing a comprehensive overview of the current state of research on the use of AI in monitoring and detecting cardiovascular events in hospital settings. 

The research conducted in May 2024 focused on literature related to the topic in review. The selection process involved developing a targeted search query, specifying the desired paper type, keywords, and subject areas. The search query was as follows: 

(“Machine Learning” OR “Data Analysis” OR “Artificial Intelligence” OR “AI” OR “Data Analytics”) AND (“Cardiovascular events”) AND (“Monitoring systems” OR “Early Detection” OR “Alert Systems”) AND (“Hospital Data” OR “Medical Data” OR “Clinical Data” OR “Physiological Data”) AND PUBYEAR>2017 AND PUBYEAR<2025 AND (LIMIT-TO (SRCTYPE , “j”)) AND (LIMIT-TO (SUBJAREA , “COMP”) OR LIMIT-TO (SUBJAREA , “ENGI”) OR LIMIT-TO (SUBJAREA , “MATE”) OR LIMIT-TO (SUBJAREA , “MATH”)) AND (LIMIT-TO (DOCTYPE , “ar”) OR LIMIT-TO (DOCTYPE , “re”)) 

The search was first applied on Scopus [12], and then, it was also attempted on the Web of Science website [13]. Textbox 1 illustrates the constructed search query, organized into sections based on keywords (Concepts, Population, Context, and Application). Additionally, specific limitations were defined, including the publication year, document type, and subject areas. It should be noted that all the papers obtained through this search query were written in English.

Textbox 1. **Process of keyword selection**

**Concepts** (7,470,936 documents)

- Machine Learning
- Data Analysis
- Artificial Intelligence
- AI
- Data Analytics

**Population** (reduced to 23,858 documents)

- Hospital Data
- Medical Data
- Clinical Data
- Physiological Data

**Context** (reduced to 1601 documents)

- Monitoring systems
- Early Detection
- Alert Systems

**Application** (reduced to 248 documents)

- Cardiovascular events

**Limitations** (reduced to 64 documents)

- Date: 2017-2024
- Document Type: Article, Reviews
- Source Type: Journal
- Limited to: Computer Science, Mathematics, Engineering

The data publication year was limited to 2017‐2024, because there were no papers, attending to the other limitations, outside of that range. This may be caused by the need to improve the health care system, combined with technological advances and available data, causing a lot of interest in this area, leading researchers to publish papers as a way of satisfying this need.

The search process yielded a progressive refinement of results, starting from an initial pool of 7,470,936 documents, then 1601 documents, culminating in a final selection of 64 relevant papers, after adding the limitations. This significant reduction reflects the effectiveness of our targeted search strategy and filtering process. The final set of 64 documents represents a focused collection of recent, relevant research in the application of AI to cardiovascular event monitoring and detection.

## Results

### Overview

**Figure 1.**
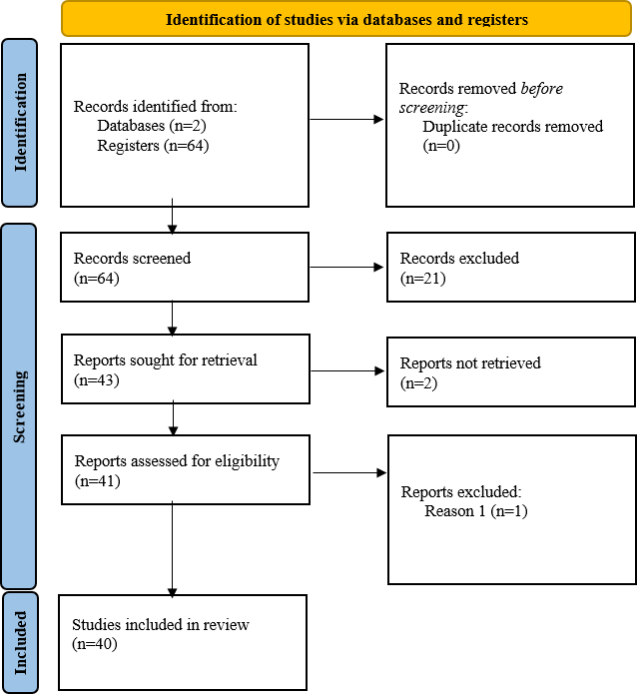
PRISMA flow diagram with the state-of-the-art results. PRISMA: Preferred Reporting Items for Systematic Reviews and Meta-Analyses.

All the papers that have been selected came from a selection process detailed in the PRISMA flow diagram (Figure 1).

The first step in the process included the removal of duplicated records, and then, a second selection was made based on the title and abstract, where 21 papers did not meet the standards. Essentially, all papers that did not focus on CVDs or events were excluded. After that, the papers that could not be retrieved were removed. In the final step, after analyzing the papers in their entirety, a selection was made, where the papers that were not relevant and that did not meet the established goal would be eliminated. In practice, it meant that any paper that did not have ML applications for the monitoring, detection or diagnosis of cardiovascular events, and diseases were excluded.

As it is shown in Figure 1, 64 documents were retrieved initially, but, after the screening, 40 studies were left in total to include. All the papers selected for this review were published between 2020 and 2024.

The remaining papers selected for inclusion were then subjected to a detailed analysis to extract data. Key themes and concepts were identified through a keyword extraction process, allowing for the categorization of papers into relevant thematic areas. This initial categorization facilitated the identification of common trends across the literature. Subsequently, a deeper analysis was conducted, focusing on the specific methodologies, objectives, results, and conclusions of each study. This in-depth examination enabled a more nuanced understanding of the research landscape, allowing for the identification of recurring approaches, significant findings, and emerging trends in the field of AI applications for CVD management. The results and discussion chapter were then constructed based on this comprehensive analysis, synthesizing the extracted data to present an overview of the current state of research in this domain.

### Outcome Analysis

#### Overview

Analyzing the application goals keywords in Table 1, the one with the biggest representation was “Early Detection” present in 31 (78%) papers, followed by “Risk Assessment,” present in 14 (35%) of the final papers. On the topics identified in the remaining 40 studies, there was a clear winner approach, as shown in Table 1: “Machine Learning” which appeared in 28 (70%) papers; however, the other techniques referenced were also related to ML, such as “Classification” and “Predictive Analysis.”

**Table 1.** Absolute frequency of the identified application goals and identified techniques within the studies included.

		Absolute frequency and percentage (n=40), n (%)
Application goals		
	Alert Systems	2 (5)
	Diagnosis	10 (25)
	Monitoring systems	11 (28)
	Risk Assessment	14 (35)
	Early Detection	31 (78)
Techniques		
	Data Analytics	3 (8)
	AI^a^	9 (23)
	Predictive Analysis	19 (48)
	Classification	24 (60)
	Machine Learning	28 (70)

a AI: artificial intelligence.

**Figure 2.**
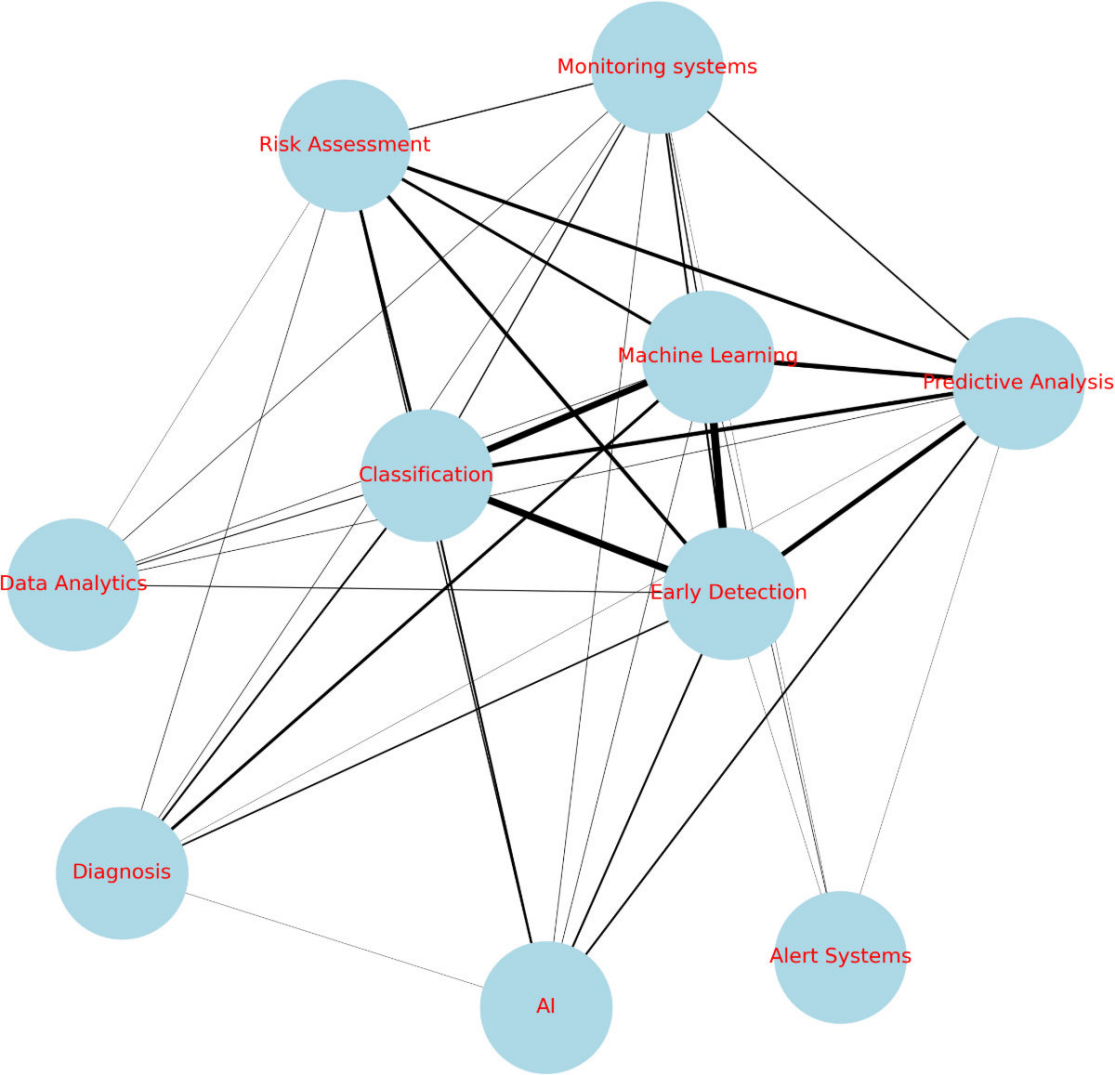
Keyword network. AI: artificial intelligence.

In Figure 2, we present a visual representation of keyword relationships constructed using Python. Our analysis focused on the existing relationships between keywords within the reviewed papers, specifically in the context of AI and cardiovascular event detection. The keyword relationships revealed in this analysis offer insights into current research trends and potential gaps in the field of AI applications for cardiovascular health.

#### Key Findings

##### Frequent Combination

As expected, the most common keyword combination across the papers is “Machine Learning” paired with “Early Detection.” This combination appears 22 times, accounting for 57.5% of the papers. This strong association underscores a significant focus on developing predictive models for timely identification of cardiovascular risks. This trend suggests a shift toward more proactive health care strategies, where AI tools could potentially identify subtle indicators of cardiovascular issues before they become clinically apparent.

##### Additional Relationships

“Classification” correlates significantly with “Early Detection” and “Machine Learning.” This could indicate an emphasis on developing nuanced categorization systems for cardiovascular conditions, potentially leading to more personalized treatment approaches.

A weaker relationship between “Predictive Analysis” and the concepts: “Machine Learning,” “Early Detection,” and “Classification” is also significant. This weaker but notable relationship hints at an emerging, more holistic approach to cardiovascular risk assessment. This could involve integrating diverse data sources and analytical methods for comprehensive, long-term health predictions.

### Future Directions

Interestingly, the relative absence of strong connections to “Alert Systems” or specific CVDs suggests areas where research might be less developed, presenting opportunities for future studies.

These insights provide context for understanding the prevailing themes in the literature but also point to potential future directions in AI-driven cardiovascular care. They suggest a trajectory toward more integrated, proactive, and personalized approaches in clinical practice, where AI tools could significantly enhance early detection, risk stratification, and treatment planning in cardiovascular medicine.

We organized the included references based on the specific topics they address, as outlined in Table 2. Recent years have witnessed significant growth in ML-based models and other innovative approaches, as evidenced by our evaluation of the selected studies.

**Table 2.** Papers by topic.

Topics	References	Papers (n=40), n (%)
Early Detection	[3,14-43]	31 (78)
Risk Assessment	[14,16,21,23,24,32,35-37,41,43-46]	14 (35)
Monitoring Systems	[17,24,35,37,39,40,46-50]	11 (28)
Diagnosis	[3,14,20,25,26,44,48,49,51,52]	10 (25)
Alert Systems	[38,50]	2 (5)

### Early Detection of CVD

#### ML Techniques

Recent studies have explored various ML techniques for classifying CVDs. Rani et al [14], Ghasemieh et al [15], Prakash and Karthikeyan [26], and Nijaguna et al [48] present different approaches, each with its own merits:

- Rani et al [14] compared several algorithms, including Naïve Bayes, support vector machine, random forest (RF), and Adaboost. The RF model achieved the best results among these.
- Ghasemieh et al [15] introduced a stacking ensemble learner (SEL) algorithm. In addition to heart-related diagnoses, SEL can be applied for COVID-19-related diagnoses.
- Prakash and Karthikeyan [26] used a more complex Dual Layer Stacking Ensemble (DLSE) classification method. This ensemble approach helps in leveraging the strengths of different models to achieve better performance.
- Nijaguna et al [48] proposed a novel approach using a Selective Opposition strategy based on artificial rabbits optimization algorithm. This novel optimization algorithm is designed to handle the high-dimensional feature space of electrocardiogram (ECG) signals.

The first two studies [14,15] reported accuracy results in the range of 86%‐88%, while the last two [26,48] demonstrated improved performance, achieving accuracy scores above 90%. This progression suggests that more sophisticated ensemble methods and optimization strategies may yield better classification results for CVD.

Another set of papers highlights the promising applications of ML for the prediction of CVD. Theerthagiri and Vidya [21] and Theerthagiri [36] focus on CVD prediction using feature elimination techniques, both achieving accuracy values of 88%. Singh et al [27] compares several traditional ML algorithms for CVD prediction such as decision trees (DT), k-nearest neighbors (KNN), artificial neural networks, and Extreme Gradient Boosting (XGBoost). Among these, XGBoost demonstrated the best performance with an accuracy of 73%. While this accuracy is lower than in some other studies, it is worth noting that the comparison of multiple algorithms provides valuable insights into their relative strengths in CVD prediction.

In a more innovative approach, Safa et al [37] introduces a hybrid clustering–based disease assessment model. This model aims to monitor patients’ cardiac and anatomic conditions, predict diseases and associated risks, and use a clustering approach to analyze patient data. The hybrid clustering–based disease assessment model achieved impressive results with prediction accuracy of up to 96% and lower error rates. This high accuracy suggests that hybrid approaches combining clustering with traditional ML techniques may offer significant improvements in CVD prediction.

Several studies focus on predicting and detecting specific cardiovascular conditions, demonstrating the versatility of ML in addressing various aspects of heart health.

Nasarian et al [19], Al-Ssulami et al [30], and Selvaraj et al [33] specifically address coronary artery disease (CAD) prediction and detection. Selvaraj et al [33] combines ML and image processing techniques for CAD prediction and detection. Using three classifiers (RF, gradient boosting tree, and multilayer perceptron [MLP]), it achieved scores higher than 95 in precision, recall, and F_1_-score. Nasarian et al [19] introduces a Heterogeneous Hybrid Feature Selection algorithm, combined with the Synthetic Minority Oversampling Technique for data balancing. This approach achieved an accuracy of 80%. Al-Ssulami et al [30] pair a ML model with an augmented dataset approach for early CVD prediction. Using a DT classifier, they achieved 99.2% accuracy with the Cleveland dataset. These exceptionally high accuracy rates suggest that data augmentation could be a powerful tool in improving ML model performance for CVD prediction.

Daliri et al [29] explores a heptagonal reinforcement learning model for predicting sinus and nonsinus arrhythmias. However, the results indicate that the model’s performance needs improvement, as the prediction accuracy was suboptimal. This highlights the challenges in applying reinforcement learning to complex medical prediction tasks and suggests areas for future research.

Itzhak et al [35] proposes a prediction model based on temporal abstraction and frequent temporal interval relational sequence patterns to predict acute hypotensive episodes. The approaches are to continuously predict “when” or “whether” an acute hypertensive episode will occur. The model’s performance to each prediction task was:

- “When” prediction: area under the receiver operating characteristic curve of 0.82, area under precision-recall curve of 0.07;
- “Whether” prediction: area under the receiver operating characteristic curve of 0.74, area under precision-recall curve of 0.71.

These results demonstrate the potential of temporal pattern analysis in predicting specific cardiovascular events, though the variation in performance metrics suggests that further refinement may be necessary.

#### DL Techniques

Recent research has explored various DL approaches for CVD classification and prediction. These studies demonstrate the potential of advanced DL techniques to significantly improve accuracy compared to conventional methods.

Dubey et al [28] and Manur et al [44] aim to classify CVD using different techniques: fuzzy deep convolutional network (FDCN) and a crow search optimization algorithm (CSOA), integrated with DL techniques, respectively. Both approaches significantly improve prediction accuracy compared to conventional classifiers, achieving scores above 95%.

Meanwhile, Nagarajan et al [52] discusses CVD classification through feature selection, using a genetic-based crow search algorithm with deep convolutional neural networks strategy. It reported an accuracy of 88.78% using all original features and 95.34% for extracted features, highlighting the critical role of feature selection and extraction in boosting the performance of DL models for classification tasks.

Abuhaija et al [41] uses various AI algorithms, such as MLP, ZeroR, and Naïve Bayes, to analyze, classify, and predict CVD. The MLP with backpropagation model achieved the highest performance, with an accuracy exceeding 95%. The MLP with backpropagation model proved to be accurate, rapid, and inexpensive medical decision-making. It has handled test datasets robustly and efficiently.

Nuryani et al [17] and Ghavidel et al [42] aim to predict and detect hypertension using convolution neural networks (CNN) and the need for cardiovascular surgery using deep neural networks, respectively. The first study achieved an F_1_-score of 98.88, while the second reached a recall of 72%, which may vary depending on the dataset used.

In contrast, Abbas et al [18] uses both ML and DL techniques to identify heart abnormalities. The study uses algorithms such as KNN, RF, XGB, a Machine and Deep Learning–based Stacked Model, and DT for heart disease prediction. Among these, the Machine and Deep Learning–based Stacked Model achieved the highest accuracy, with a score of 94.14%. By gathering and growing more data, in this case, DL techniques better handled the prediction problem and produced better results.

#### Reviews

Parashar et al [31] explores the current state-of-the-art application of ML in the detection, categorization, and prediction of CVD and respiratory diseases. Key findings include the prevalent use of clinical features such as age, blood pressure (BP), and cholesterol for CVD prediction, and the dominance of CNN for feature selection. Challenges identified include the need for standardized frameworks, data privacy, and algorithm interpretability.

In Rani et al [3], researchers conclude that combining various ML algorithms (such as RF, support vector machine, XGBoost, AdaBoost, deep neural network or CNN, and KNN) with expert cardiologist input on feature prioritization and leveraging unstructured health care data can significantly enhance the accuracy of heart disease prediction systems. Collectively, these studies underscore the transformative potential of AI and ML in improving CVD classification accuracy.

### Diagnosis of CVD

Some papers aim specifically at the diagnosis of these kinds of diseases. Al-Absi et al [20] presents a DL approach using the retinal image and dual-energy x-ray absorptiometry to diagnose CVD, achieving an accuracy of 78.3% in distinguishing between patients with CVD and controls. The findings suggest that integrating retinal images with dual-energy x-ray absorptiometry data improves the accuracy of CVD detection, while also highlighting the greater discriminatory power of fat content and bone mineral density over muscle mass and bone areas for CVD diagnosis. Chen et al [38] used self-supervised learning to pretrain generalized laboratory progress model with the aim of detecting CVD, which significantly improves the classification accuracy from 0.63 to 0.90. The results reveal that linear and Piecewise Cubic Hermite Interpolating Polynomial interpolation enhances self-supervised learning performance, by expanding the tolerance for periods of absence. This allows for less frequent patient returns and examinations, reducing travel and medical expenses, and suggests that patients can benefit from diminished frequency of visits and onerous examinations through sustainable estimation methods.

Also, Kakudi et al [25] explores ML methodologies for the diagnosis of metabolic syndrome (MetS). The findings provide valuable insights for researchers and health care practitioners into current trends and developments, suggesting that nonclinical methods could serve as early preventive tools for managing MetS and CVD.

### Monitoring Patients

A certain number of publications focus on the use of ECG, BP, and wearable devices for monitoring cardiovascular health. Širaiy et al [39], Charlton et al [40], and Alugubelli et al [46] center around the use of wearable technology for monitoring cardiovascular health. These papers emphasize the role of wearable technology in providing continuous, noninvasive monitoring of cardiovascular parameters, contributing to early detection and management of heart conditions. Lyon et al [22] provides a literature review that describe the clinical applications and main ML methods currently used for ECG analysis, and Agham and Chaskar [49] provides a perspective of ML approaches using cuffless BP for the generation of predictive models for continuous BP measurement. Pires et al [50] also aims to develop innovative solutions to empower patients with CVD, by providing them with personalized plans for treatment and increasing their ability for self-monitoring.

Varma et al [47] aimed to evaluate the current status of mobile health (mHealth) technologies, including AI-driven tools, in the monitoring and management of heart rhythm disorders. The paper reviewed various mHealth modalities, such as wearable devices, smartphone-based ECG, and photoplethysmography, and discussed their potential in detecting atrial fibrillation and other arrhythmias. The authors highlighted the role of predictive analytics in improving clinical decision-making and patient self-management. Despite the promising applications, the study noted several challenges, including data validation, integration into clinical practice, and cybersecurity concerns. The statement concluded that while mHealth holds significant potential for enhancing CVD management, particularly in arrhythmia detection, further research is needed to overcome operational barriers and optimize its integration into health care systems.

### Risk Assessment

The papers presented in this paragraph collectively emphasize the application of advanced ML and AI techniques to improve CVD risk assessment and prediction. Faizal et al [16] and Jamthikar et al [45] highlight the superior performance of AI-based predictive models compared to traditional methods like the Global Registry of Acute Cardiac Events and thrombolysis in myocardial infarction, with AI frameworks incorporating noninvasive data for enhanced accuracy. Groccia et al [23] demonstrates the effectiveness of cost-sensitive models in predicting cardiovascular deterioration in patients with chronic heart failure, stressing the clinical importance of early detection. This last system achieved sensitivity scores of 65% and specificity scores of 55%. Similarly, Bostani et al [32] introduces a novel method combining reinforcement learning and MLPs for early risk detection in athletes, achieving an *F*_1_-measure of 87.4% and a geometric mean of 89.6%. Navarrete et al [43] confirms that RF models perform well in predicting mortality risk in older adults, suggesting practical applications in health care settings. Gunasekaran et al [34] presents an Internet of Health care Things–integrated model that improves accuracy in CVD prediction, enhancing performance and data security. The presented model achieved an impressive accuracy of 97%. These findings collectively underscore the potential of AI and ML in transforming CVD risk prediction and management, providing more accurate, early, and cost-effective solutions.

Baek and Arzani [24] and Kadem et al [51] emphasize the transformative potential of integrating hemodynamic modeling, medical imaging, and ML in understanding cardiovascular anomalies and managing abdominal aortic aneurysms. Both highlight the importance of personalized medicine and data-driven approaches, particularly through physics-based ML models, in improving diagnostic accuracy and risk assessment. They address the need for extensive multisite and multimodal patient studies to validate these tools’ benefits, accuracy, and safety. As computational power and data availability increase, these technologies are expected to enhance patient outcomes and reduce health care costs related to CVDs.

## Discussion

### Principal Findings

The literature review reveals significant advancements in the use of ML and DL techniques for the classification, prediction, and diagnosis of CVD and events. Techniques such as DLSE, FDCN, and CSOA have demonstrated high accuracy, surpassing 95%. Feature selection and elimination methods like Selective Opposition strategy based on artificial rabbits optimization, genetic-based crow search algorithm, and recursive feature elimination (RFE)–based gradient boosting further enhance prediction accuracy.

Some of the studies aim to predict the detection of CVD while others specify their ML algorithms to predict and manage specific conditions such as CAD, arrhythmia, hypertension, MetS, and abdominal aortic aneurysms.

Various studies highlight the importance of integrating multiple data sources and expert input to improve model performance. Wearable technologies and noninvasive methods are emphasized for continuous monitoring and early detection, showing promise in reducing health care costs and patient burden. Additionally, the application of AI in predicting emergency readmissions and the need for cardiovascular surgery underscores its potential to improve patient outcomes. Challenges identified include the need for standardized frameworks, data privacy, and algorithm interpretability.

Multimedia Appendix 1 provides a summary of the various AI methodologies and datasets used in CVD monitoring and prediction. The advantages of ensemble techniques like the SEL and the DLSE method lie in their ability to combine multiple models for improved accuracy, as evidenced by results ranging from 88% to over 95% accuracy. However, their complexity and interpretability present significant challenges that could hinder their adoption in clinical settings. Similarly, techniques like RFE and gradient boosting highlight the importance of feature selection in enhancing model precision, but the imbalanced nature of datasets used in some studies, such as the Nasarian CAD dataset, introduces limitations in the generalizability of results. The current literature often focuses on performance metrics like accuracy, precision, and *F*_1_-score, without offering a robust analysis of the practical limitations of these systems, such as computational cost, scalability, or real-world clinical applicability.

The most frequently used datasets across the reviewed literature include the Cleveland Heart Disease dataset and the Cardiovascular Disease dataset. Some studies leverage datasets from Kaggle, which offer larger, publicly available cardiovascular data that span a variety of CVD conditions. These datasets are valuable for training more generalizable models, but they often demonstrate class imbalances, as seen in studies using RFE and gradient boosting.

Overall, these findings collectively underscore the potential of AI and ML in enhancing CVD risk assessment, prediction, and management. The application of advanced methodologies such as ensemble learning and optimization strategies offers more accurate and early detection solutions, as evidenced by performance improvements across various datasets. However, the limitations in dataset diversity, model interpretability, and scalability highlight the ongoing need for more comprehensive real-world validations. Additionally, integrating discussions on the specific advantages, disadvantages, and performance measures of existing systems will further strengthen the understanding of their practical applications. Future research should focus on overcoming these challenges to develop more robust, cost-effective, and generalizable AI-driven solutions for cardiovascular health care.

### Future Work

The future research prospects, across the studies referenced, suggest promising opportunities for integrating advanced technologies to improve CVD management. Combining multiomics data (genomics, proteomics, etc) with AI and Internet of Things could potentially enhance precision in disease detection, decision-making, and personalized care. Developing hybrid ML models using real-time and large datasets might improve heart disease prediction. Computational techniques for ECG analysis show the potential to uncover new biomarkers and enhance patient risk stratification. Wearable devices and remote monitoring systems could transform patient care by providing continuous, real-time health data. Addressing challenges such as data quality, standardization, and regulatory approval will be necessary to advance these innovations. Additionally, incorporating nonclinical models, evaluating diverse datasets, and leveraging advanced computational resources may be beneficial for developing effective and scalable solutions.

### Conclusions

This literature review underscores the transformative potential of AI and ML in revolutionizing cardiovascular health care. Advanced AI-driven methodologies, including DLSE, FDCN, and CSOA, have demonstrated remarkable improvements in the detection, prediction, and management of CVD.

The integration of diverse data sources with these sophisticated algorithms enhances diagnostic accuracy, supports personalized treatment strategies, and enables early intervention efforts. Furthermore, the application of AI in wearable technologies shows great promise for continuous, noninvasive health monitoring, potentially improving patient outcomes while reducing health care costs.

These advancements represent a significant shift toward more proactive and personalized patient care, offering health care professionals powerful tools to support their decision-making processes. The high accuracy demonstrated by AI models in CVD classification and prediction suggests a future where cardiovascular events can be anticipated and prevented with greater precision.

However, the widespread adoption of AI in health care faces several challenges. These include the standardization of frameworks and protocols, ensuring robust data privacy and security measures, enhancing the interpretability and explainability of AI algorithms, validating AI models across diverse patient populations, and addressing ethical considerations in AI-driven health care.

Overcoming these obstacles could be crucial to realizing the full potential of AI-driven technologies in transforming cardiovascular care. Future research should focus on: (1) refining and optimizing AI models for improved accuracy and reliability; (2) integrating real-time data streams to enhance the applicability of AI in clinical settings; (3) conducting large-scale, multicenter studies to validate AI efficacy across different health care systems and patient demographics; (4) developing guidelines and best practices for the ethical implementation of AI in health care; and (5) exploring the synergies between AI and other emerging technologies to create more comprehensive health care solutions.

By addressing these challenges and pursuing these research directions, AI and ML have the potential to revolutionize cardiovascular health care on a global scale. The promise of more accurate, timely, and cost-effective solutions could significantly benefit both patients and health care providers, ultimately leading to improved cardiovascular health outcomes.

As we move forward, it is essential to maintain a balance between technological innovation and ethical considerations, ensuring that the advancements in AI-driven cardiovascular care are accessible, equitable, and centered on improving patient well-being. The integration of these technologies into clinical practice represents a significant step toward more proactive, personalized, and efficient cardiovascular care, aiming to improve global health outcomes.

## Supplementary material

10.2196/64349 Multimedia Appendix 1 Summary of the paper’s methods, used datasets, limitations, and performance measures.

10.2196/64349 Checklist 1 PRISMA-ScR checklist.

## Abbreviations

AI: artificial intelligence
BP: blood pressure
CAD: coronory artery disease
CNN: convolution neural network
CSOA: crow search optimization algorithm
CVD: cardiovascular diseases
DL: deep learning
DLSE: Dual Layer Stacking Ensemble
DT: decision tree
ECG: electrocardiogram
FDCN: fuzzy deep convolutional network
KNN: k-nearest neighbor
MetS: metabolic syndrome
mHealth: mobile health
ML: machine learning
MLP: multilayer perceptron
PRISMA: Preferred Reporting Items for Systematic Reviews and Meta-Analyses
RF: random forest
RFE: recursive feature elimination
SEL: stacking ensemble learner
XGB: Extreme Gradient Boosting

## References

[1] Kagiyama N, Shrestha S, Farjo PD, Sengupta PP (2019). Artificial intelligence: practical primer for clinical research in cardiovascular disease. J Am Heart Assoc.

[2] Habehh H, Gohel S (2021). Machine learning in healthcare. Curr Genomics.

[3] Rani P, Kumar R, Jain A (2024). An extensive review of machine learning and deep learning techniques on heart disease classification and prediction. Arch Computat Methods Eng.

[4] Elvas LB, Nunes M, Ferreira JC, Dias MS, Rosário LB (2023). AI-driven decision support for early detection of cardiac events: unveiling patterns and predicting myocardial ischemia. J Pers Med.

[5] Ogunpola A, Saeed F, Basurra S, Albarrak AM, Qasem SN (2024). Machine learning-based predictive models for detection of cardiovascular diseases. Diagnostics (Basel).

[6] Sun X, Yin Y, Yang Q, Huo T (2023). Artificial intelligence in cardiovascular diseases: diagnostic and therapeutic perspectives. Eur J Med Res.

[7] Cardiovascular diseases. WHO.

[8] Cardiovascular diseases (CVDs). WHO.

[9] Cardiovascular disease burden. Pan American Health Organization.

[10] Gala D, Behl H, Shah M, Makaryus AN (2024). The role of artificial intelligence in improving patient outcomes and future of healthcare delivery in cardiology: a narrative review of the literature. Healthcare (Basel).

[11] PRISMA statement. https://www.prisma-statement.org.

[12] Scopus Preview.

[13] Web of Science.

[14] Rani P, Kumar R, Ahmed N, Jain A (2021). A decision support system for heart disease prediction based upon machine learning. J Reliable Intell Environ.

[15] Ghasemieh A, Lloyed A, Bahrami P, Vajar P, Kashef R (2023). A novel machine learning model with Stacking Ensemble Learner for predicting emergency readmission of heart-disease patients. Decis Anal J.

[16] Faizal ASM, Thevarajah TM, Khor SM, Chang SW (2021). A review of risk prediction models in cardiovascular disease: conventional approach vs. artificial intelligent approach. Comput Methods Programs Biomed.

[17] Nuryani N, Utomo TP, Prabowo NA, Yazid M, Yunianto M (2024). Advancing non-cuff hypertension detection: leveraging 1D convolutional neural network and time domain physiological signals. Int J Onl Eng.

[18] Abbas S, Sampedro GA, Alsubai S, Almadhor A, Kim T hoon (2023). An efficient stacked ensemble model for heart disease detection and classification. Comput Mater Contin.

[19] Nasarian E, Abdar M, Fahami MA (2020). Association between work-related features and coronary artery disease: a heterogeneous hybrid feature selection integrated with balancing approach. Pattern Recognit Lett.

[20] Al-Absi HRH, Islam MT, Refaee MA, Chowdhury MEH, Alam T (2022). Cardiovascular disease diagnosis from DXA scan and retinal images using deep learning. Sensors (Basel).

[21] Theerthagiri P, Vidya J (2022). Cardiovascular disease prediction using recursive feature elimination and gradient boosting classification techniques. Expert Syst.

[22] Lyon A, Mincholé A, Martínez JP, Laguna P, Rodriguez B (2018). Computational techniques for ECG analysis and interpretation in light of their contribution to medical advances. J R Soc Interface.

[23] Groccia MC, Guido R, Conforti D (2023). Cost-sensitive models to predict risk of cardiovascular events in patients with chronic heart failure. Information.

[24] Baek S, Arzani A (2022). Current state-of-the-art and utilities of machine learning for detection, monitoring, growth prediction, rupture risk assessment, and post-surgical management of abdominal aortic aneurysms. Appl Eng Sci.

[25] Kakudi HA, Kiong LC, Moy FM, Kau LC, Pasupa K (2021). Diagnosis of metabolic syndrome using machine learning, statistical and risk quantification techniques: a systematic literature review. Malays J Comput Sci.

[26] Prakash VJ, Karthikeyan NK (2022). Dual-layer deep ensemble techniques for classifying heart disease. Inf Technol Control.

[27] Singh E, Singh V, Rai A, Christopher I, Mishra R, Arikumar KS (2024). Early detection of cardiovascular disease with different machine learning approaches. EAI Endorsed Trans IoT.

[28] Dubey AK, Sinhal AK, Sharma R (2024). Heart disease classification through crow intelligence optimization-based deep learning approach. Int J Inf Technol.

[29] Daliri A, Sadeghi R, Sedighian N, Karimi A, Mohammadzadeh J (2024). Heptagonal Reinforcement Learning (HRL): a novel algorithm for early prevention of non-sinus cardiac arrhythmia. J Ambient Intell Human Comput.

[30] Al-Ssulami AM, Alsorori RS, Azmi AM, Aboalsamh H (2023). Improving coronary heart disease prediction through machine learning and an innovative data augmentation technique. Cogn Comput.

[31] Parashar G, Chaudhary A, Pandey D (2024). Machine learning for prediction of cardiovascular disease and respiratory disease: a review. SN Comput Sci.

[32] Bostani A, Mirzaeibonehkhater M, Najafi H (2023). MLP-RL-CRD: diagnosis of cardiovascular risk in athletes using a reinforcement learning-based multilayer perceptron. Physiol Meas.

[33] Selvaraj R, Satheesh T, Suresh V, Yathavaraj V (2023). Optimized machine learning for CHD detection using 3D CNN-based segmentation, transfer learning and adagrad optimization. SSRG Int J Electr Electron Eng.

[34] Gunasekaran K, Kumar VDA, Jayashree K (2024). Prediction and risk analysis of cardio vascular diseases in IoHT by enhanced CHIO-based residual and dilated gated network with attention mechanism. Biomed Signal Process Control.

[35] Itzhak N, Pessach IM, Moskovitch R (2023). Prediction of acute hypertensive episodes in critically ill patients. Artif Intell Med.

[36] Theerthagiri P (2022). Predictive analysis of cardiovascular disease using gradient boosting based learning and recursive feature elimination technique. Intell Syst Appl.

[37] Safa M, Pandian A, Gururaj HL, Ravi V, Krichen M (2023). Real time health care big data analytics model for improved QoS in cardiac disease prediction with IoT devices. Health Technol.

[38] Chen LC, Hung KH, Tseng YJ (2024). Self-supervised learning-based general laboratory progress pretrained model for cardiovascular event detection. IEEE J Transl Eng Health Med.

[39] Širaiy B, Ilić V, Toskić L (2021). Usability of wireless ECG body sensor for cardiac function monitoring during field testing. Meas Sci Rev.

[40] Charlton PH, Kyriaco PA, Mant J, Marozas V, Chowienczyk P, Alastruey J (2022). Wearable photoplethysmography for cardiovascular monitoring. Proc IEEE Inst Electr Electron Eng.

[41] Abuhaija B, Alloubani A, Almatari M (2023). A comprehensive study of machine learning for predicting cardiovascular disease using Weka and Statistical Package for Social Sciences tools. Int J Electr Comput Eng.

[42] Ghavidel A, Pazos P, Del Aguila Suarez R, Atashi A (2024). Predicting the need for cardiovascular surgery: a comparative study of machine learning models. J Electron Electromed Eng Med Inform.

[43] Navarrete JP, Pinto J, Figueroa RL, Lagos ME, Zeng Q, Taramasco C (2022). Supervised learning algorithm for predicting mortality risk in older adults using cardiovascular health study dataset. Appl Sci (Basel).

[44] Manur M, Pani A, Kumar P (2021). A big data analysis using fuzzy deep convolution network based model for heart disease classification. Int J Intell Eng Syst.

[45] Jamthikar AD, Gupta D, Saba L (2020). Artificial intelligence framework for predictive cardiovascular and stroke risk assessment models: A narrative review of integrated approaches using carotid ultrasound. Comput Biol Med.

[46] Alugubelli N, Abuissa H, Roka A (2022). Wearable devices for remote monitoring of heart rate and heart rate variability-what we know and what is coming. Sensors (Basel).

[47] Varma N, Cygankiewicz I, Turakhia MP (2021). 2021 ISHNE/HRS/EHRA/APHRS collaborative statement on mHealth in arrhythmia management: digital medical tools for heart rhythm professionals: from the International Society for Holter and Noninvasive Electrocardiology/Heart Rhythm Society/European Heart Rhythm Association/Asia Pacific Heart Rhythm Society. Cardiovasc Digital Health J.

[48] Nijaguna GS, Lal ND, Divakarachari PB, Prado RP de, Woźniak M, Patra RK (2023). Feature selection using selective opposition based artificial rabbits optimization for arrhythmia classification on internet of medical things environment. IEEE Access.

[49] Agham ND, Chaskar UM (2021). Learning and non-learning algorithms for cuffless blood pressure measurement: a review. Med Biol Eng Comput.

[50] Pires IM, Denysyuk HV, Villasana MV (2021). Mobile 5P-medicine approach for cardiovascular patients. Sensors (Basel).

[51] Kadem M, Garber L, Abdelkhalek M, Al-Khazraji BK, Keshavarz-Motamed Z (2023). Hemodynamic modeling, medical imaging, and machine learning and their applications to cardiovascular interventions. IEEE Rev Biomed Eng.

[52] Nagarajan SM, Muthukumaran V, Murugesan R, Joseph RB, Meram M, Prathik A (2022). Innovative feature selection and classification model for heart disease prediction. J Reliable Intell Environ.

