# Supplementary material for: The Role of AI in Cardiovascular Event Monitoring and Early Detection: Scoping Literature Review
Source: JMIR Med Inform. 2025 Mar 6;13:e64349. doi: 10.2196/64349 (PMC11905924; doi:10.2196/64349)
Supplement: Multimedia Appendix 1 [file medinform-v13-e64349-s001.docx]

|  | **Method** | **Dataset** | **Limitations** | **Performance measures** |
| --- | --- | --- | --- | --- |
| **Studies** |  |  |  |  |
| [15] | Classification using traditional methods | Cleveland Heart disease dataset | - Needs Improvement | Accuracy: 86.6% |
| [16] | SEL algorithm | Private dataset from the MIT Laboratory for Computational Physiology | - Complexity - Interpretability | F1-Score: 0.84; AUC: 88%; Accuracy: 88% |
| [20] | 2HFS algorithm | Nasarian CAD dataset | - Small Dataset Size - Complexity | Accuracy: 81.2%; Precision: 80.2%; Recall: 85.2%; F-score: 82% |
| [21] | DXA Model, Retinal Image Model and Hybrid Model | De-identified dataset from (QBB) | - Limited Generalizability - Potential Bias | Accuracy: 78.3% |
| [22] | Recursive feature elimination and gradient boosting technique | Cardiovascular disease dataset from Kaggle | - Complexity - Imbalance in dataset | Accuracy: 89.8% |
| [24] | SVMs, ANN, NB, DT, and RF, applying a cost-sensitive method | Dataset constructed from real data | - Limited Dataset Size - Imbalanced Dataset - Complexity | Sensitivity: 65%; Specificity: 55%; mean area under the curve: 0.71 |
| [27] | DLSE method | Stalog Dataset; SPECTF Dataset; SPECT Dataset; Eric Heart Dataset; NHANES coronary heart disease Dataset | - Complexity - Computational Cost - Interpretability | Accuracy: 95.2% |
| [28] | DT, XGBoost, KNN, and ANN | Dataset from Kaggle but not specified | - Scalability - Computational Cost | Accuracy: 73.4% |
| [29] | CSOA for feature selection and DNN and CNN for classification | The Cardio dataset and the NIH chest X-ray image dataset | - Limited Generalizability - Scalability Issues - Computational Cost | Accuracy: 96% |
| [30] | HRL algorithm | 12-lead electrocardiogram (ECG) database | - Interpretability - Complexity | (not provided by the authors) |
| [31] | Paired machine learning model with an augmented dataset approach | Comprehensive heart disease dataset | - Limited Dataset Size - Complexity | Accuracy: 99.2% |
| [33] | Reinforcement Learning (RL) based multilayer perceptron, termed MLP-RL-CRD | Assembled dataset containing medical records from athletes | - Complexity - Implementation | F-measure: 87.4%; Geometric mean: 89.6% |
| [34] | 3D CNN based Segmentation technique | Dataset of sample images collected from patients with and without coronary heart disease (CHD) | - Data Privacy and Security Concerns - Generalizability | Precision, Recall and F-Measure score higher that 95% |
| [35] | Enhanced Coronavirus Herd Immunity Optimizer with Adaptive Residual and Dilated Long Short Term Memory and Attention Mechanism (ECHIO-ARDL-AM) model | Heart Disease Cleveland UCI dataset | - High Computational Power - Security Concerns | Accuracy: 97% |
| [36] | Prediction model based on temporal abstraction and frequent time-intervals-related patterns (TIRPs) | Medical Information Mart for Intensive Care (MIMIC-III) database. | - Imbalanced Data - Computational Complexity - Generalizability | ‘‘when’’ approach:  AUC-ROC: 0.82, AUPRC: 0.07;  ‘‘whether’’ approach: AUC-ROC: 0.74, AUPRC: 0.71. |
| [37] | RFE-GB approach | Cardiovascular Disease Dataset from Kaggle | - Complexity - Dataset Imbalance | Accuracy: 88.8%; Precision: 88%; Recall: 85%; F1-Score: 83% |
| [38] | HCBDA model | Real-time health care big data obtained via Internet of Things (IoT) devices attached to patients | - Imbalance of classes | Accuracy: up to 96% |
| [39] | GLP model | Pretext Dataset, sourced from the Chang Gung Research Database and Downstream Dataset, obtained from Taipei Veterans General Hospital | - Computational Cost - Interpretability | Accuracy: 90% |
| [42] | Weka Workbench, using ZeroR, NB, KNN, Logistic Regression, J48, SVM, MLP | Collected medical dataset | - Complexity - Needs Optimization - Limited Generalization | Sensitivity: 95.1%; Specificity: 90%; Precision: 95.1%; Accuracy: 95.1% |
| [43] | Machine Learning (ML) models, mainly focusing on Deep Neural Networks (DNNs) | Dataset from hospitals affiliated with Shahid Beheshti University of Medical Sciences and Health Services in Iran | - Generalizability - Complexity - Data Privacy Concerns | Recall: 72% |
| [44] | Employed three machine learning models: RF, SVM, and logistic regression | Cardiovascular Health Study (CHS) dataset | - Complexity - Generalizability Issues | Accuracy: 89% |
| [45] | FDCN model | Medical data acquired from Cleveland heart disease UCI datasets | - Complexity - Limited Generalization - Optimization Needs | Precision: 89.5%; Recall: 94.8%; F-score: 92.0%; Accuracy: 95.6% |
| [49] | Selective  Opposition strategy based Artificial Rabbits Optimization (SOARO) for selecting the optimal feature subset | MIT-BIH arrhythmia dataset | - Complexity - Scalability Issues | Accuracy: 98.9% |
| [51] | 5P (Predictive, Preventive, Participatory, Personalized, and Precision) medicine approach | Data gathered using sensors, including inertial, acoustic, and imaging sensors. | - Technical Challenges (reliability) | (Not provided by the authors) |
| [53] | GCSA with DCNN approach | Used ten different medical datasets: dermatology dataset, heart-C dataset, lung cancer dataset, pima Indian dataset, hepatitis disease dataset, Iris disease dataset, Wisconsin cancer dataset, Lymphography dataset, diabetes disease dataset and  Statlogheart disease dataset. | - Complexity - Interpretability | Accuracy: 95.3% |
